# Incompleteness of urban infrastructures in transition: Scenarios from the mobile age in Nairobi

**DOI:** 10.1177/0306312720927088

**Published:** 2020-05-26

**Authors:** Prince K Guma

**Affiliations:** Human Geography and Spatial Planning, Utrecht University, Netherlands

**Keywords:** African technologies, heterogeneity, incompleteness, mobile age, urban infrastructure

## Abstract

Work in policy and research circles tends to depict urban infrastructural heterogeneity as synonymous with failure or brokenness. Inherent in this tendency is the often-subtle expectation that infrastructures should evolve as do their counterparts elsewhere, or in a linear trajectory from less complete to more complete arrangements. This article opposes such completist lures and inclinations. I recuperate the notion of incompleteness as a constitutive feature and explanatory category for urban infrastructures that, while diverging from so-called norms and ideals, cannot be described as failed or broken. I argue that, rather than devising universalizing solutions to processes of infrastructural heterogeneity, it is perhaps better to see infrastructures as emergent, shifting and thus incomplete. I make this case looking at three successive infrastructures in Nairobi: the Simu ya Jamii kiosk, the M-Pesa stall and the M-Pesa platform. I examine these infrastructures not simply as raw materials or empirical conduits, but as the very starting point in theorizing urban infrastructures from the South. Ultimately, this study not only opens up a vital frame for situated analysis and understanding of urban infrastructures in transition, it also adds to and extends STS analytical frames into non-Northern contexts.

## Introduction

Since launching its operations in October 2000, Safaricom, Kenya’s leading mobile service operator, has capitalized on unique modes of service provision in the East African region. For example, it has pioneered a facility popularly known in Kenya’s daily parlance as ‘Simu ya Jamii’ (Swahili for communal phone), namely a cordless and community-based pay phone typically housed in a container-like structure configured in roughly the shape and size of a phone booth, and attended by an agent. It has also established the world’s foremost mobile money service, known as ‘M-Pesa’ (M stands for Mobile and Pesa is Swahili for Money), which facilitates money transfers and payments, and also serves as a virtual depository of cash and intermediate infrastructure for microfinance disbursement and remittance deliveries. M-Pesa – like Simu ya Jamii at its height – functions through strong agent networks facilitated by mundane and ephemeral structures known as ‘kiosks’ or ‘stalls’. These infrastructure developments have come to constitute the matrix of the mobile age in Kenya. They are noticeably temporary, ad hoc and makeshift, and often diverge from standard expectations of how mobile technologies ought to evolve, function or look like. Hence, they are what could be understood as ‘heterogeneous’ infrastructure. But they are also more than just that: They are malleable, create possibilities in their ephemerality and exhibit a tendency to be and remain incomplete. As such, they trigger new ways of thinking about urban infrastructures in transition.

The aim of this article, therefore, is to think anew, through the lens of incompleteness, processes of infrastructural heterogeneity and diversity. I use the notion of incompleteness in a pluralistic sense; where it is grounded in a restrained form of relativism, beyond totalistic accounts. Moreover, I employ the notion under a non-binary approach where ‘incompleteness’ is not simply the opposite of completeness. Completeness and incompleteness as normative ways of being are intricately co-constituted and exist simultaneously. Thus, my use of the notion without any brackets, slash, or cut or void at the center is deliberate. I make the case for incompleteness as a notion that opens up a frame for analysis of a kind of urban infrastructures that, while diverging from so-called norms and ideals, cannot be described as failed or broken, but as something else entirely. I investigate ‘incompleteness’ as an explanatory category for infrastructures that do not yield to general standards, and also as a corrective to dominant intellectual loops and circuits that regard everything that does not appear to conform as failed.

Recognizing incompleteness displays the fact that infrastructure is always in the making, and therefore incomplete. Likewise, it forecloses frames that see heterogeneous infrastructures in the South as defective or symbolic of a pathology of the under-developed world. Such frames not only fall short over what exactly constitutes failure or deficiency, but also barely provide an understanding of infrastructural heterogeneity and diversity. Hence, I recognize successive periods in the development and growth of mobile telephony in Nairobi, employing *transiency* (the state of lasting only a short time), *continuity* (the incessant maintenance of continuous operations over time) and *contingency* (the capability of systems to create possibilities amid uncertainty) as ‘elements of incompleteness’ to make salient the nature of infrastructure as inherently incomplete.

The article is based on an empirical study of the spread of mobile infrastructures in Nairobi, the capital of Kenya. Rather than doing a comprehensive or exhaustive examination of a single case (i.e. Safaricom) or sector (i.e. telecommunications), I examine small and marginal infrastructures of the mobile age that remain relatively peripheral in theorizing infrastructures within critical STS and urban studies.^1^ These infrastructures illuminate the inherent nature of incompleteness. I take these infrastructures as technical objects,^2^ as material things and ‘as life systems with their own motility’ (Amin and Thrift, 2017: 122). I examine them not simply as raw materials or empirical conduits but as entry points to theorizing urban infrastructures from the South. This is imperative since the hegemonic theorization on urban infrastructure and technological innovation has long been the preserve of North America and Western Europe. With a few exceptions (e.g. Mavhunga, 2014, 2017), intellectual theorizations are offered by scholars from – or oriented within the well-worn paths of – the Global North. More often than not, intellectual loops and circuits in the form of indigenous knowledges and theories from the South are either disparaged, denigrated, deflated or marginalized as alternative curiosities or merely belief systems not worthy of mainstream analysis (see e.g. Mavhunga, 2014). As such, circuits of knowledge out of the South are often made to lie out of the mainstream – that is, ‘anthropologized or used as footnote fodder in the western academy’ (Seth et al., 1998: 10). Emergent scholars are sometimes left with no choice but to reiterate North-oriented constructs, while using African spaces and places as laboratories or test grounds for such constructs sometimes without due regard for local realities and patterns of thought, practice and ingenuity.

I enrich infrastructure studies with work that draws from located and situated conceptualizations in and of the South. I ask: How might studying and theorizing through African technological infrastructures like Simu ya Jamii and M-Pesa illuminate and reframe the way we think about infrastructures in transition? What do these infrastructures add to extant analytical constructs in STS studies?^3^ In grappling with these questions, I bring critical STS, urban studies, and Africanist philosophy into dialogue.

Methodologically, I ground my analysis in empirical qualitative methods drawing from primary observations and interviews based on ongoing research in Nairobi since January 2015. In my field visits, I focused on a situated exploration, particularly the urban areas of Kibera, Mathare and Soweto Kayole. These spaces were specifically chosen for three reasons. First, they accommodated different variants of the infrastructures of interest to this study. Second, at the fringes of Nairobi, they have been a key demographic in the mobile telecommunications market space in part as significant spaces of technological appropriation and experimentation. And third, as some of the most ordinary urban areas, these spaces have come to define Nairobi’s expanse as one synonymous with diverse and heterogeneous infrastructure. During my time in these spaces, I kept a journal for interesting observations and fieldnotes, and took photographs of interesting infrastructural and technological developments. I interviewed 35 insiders in the city, all with different forms of expertise and knowledge about the workings, histories and patterns of the infrastructures in question. These included urban residents, community leaders and representatives, bloggers and journalists, technology developers and experts, digital strategists, and mobile service providers. The interviews were mostly conversational in style and semi-structured. The opinions and insights deriving from these conversations were imperative not only for substantiating specific scenarios in the growth and development of mobile telephony in Nairobi, but also for discerning how inside observers and actors perceive and interpret the very nature of emergent infrastructures of the mobile age. These opinions and insights were important for demonstrating how and in what forms incompleteness (of infrastructure) is or becomes salient for insiders in a Southern city.

## Disarticulating ‘incompleteness’ for the study of infrastructure

### Countering completist pursuits in STS and urban studies

While infrastructure has long been an ideal subject of technical studies, it is only relatively recently that the topic has attracted broader and more dynamic attention, not least within the social sciences, including geography, anthropology, philosophy, sociology, architecture, planning and history. In STS, the discourse around urban infrastructure can be traced to the large technical systems (LTS) paradigm introduced in 1987 by Hughes (e.g. 1987). This paradigm, which focuses on centralized and extensive technological networks like telecommunications, transport, electricity, waste, and water and sanitation networks, provides the basis upon which contemporary infrastructure studies have materialized. It has ignited constructive debate founded on the critique of its limitations in thinking about urban infrastructures in transition (Bowker et al., 2009; Graham, 2010; Moss, 2016; Star, 1999). For instance, it has been criticized for assuming entrenched universality of a single or dominant infrastructure network, viewing some infrastructures as closed-ended and complete and others as open-ended and incomplete. It also has been criticized for suggesting an illusory one-size-fit-all view in which infrastructures that do not yield or conform to universality are explicitly disparaged as failed, broken or lying outside of the norm.

The seminal work of Star and Ruhleder (1994, 1996) offers a more explicit framework for conceptualizing infrastructure as a fundamentally contextualized relation rather than a thing or set of things. Star and Ruhleder pose the question, ‘when is an infrastructure?’ (1996: 112), inciting work and interest about when it is exactly that an object becomes a practice. This provocation has contributed toward defining infrastructure as relational, and for extending the conceptual reach of infrastructure studies to the social and cultural. In other words, this work has inspired a constructivist understanding of infrastructures, emerging ‘for people in practice, connected to activities and structures’ (Star and Ruhleder, 1996: 112).

In the past two decades, scholars have called for the fusion of STS studies of infrastructure with urban studies. Of particular note, Hommels (2005) encourages us to transcend the sociotechnical status quo of the urban sphere as static and invariable, and to pay more attention to its obduracy and constantly changing reality. This call is furthered by Furlong (2011), who reasons that while geography and STS both reveal important concerns, they posit different vantage points and overlook each other’s. This, Furlong argues, is sufficient motive for us to be more open to integrating and coalescing these fields. Following these calls, the intellectual discourse around the study of infrastructure has evolved (Coutard and Guy, 2007; Graham 2010), with a number of disciplines beginning to converge. One interesting example is by Carse and Kneas (2019), who speak to a provocative question initially raised by Star (1999: 378): ‘When is an infrastructure finished, and when would we know that?’ Carse and Kneas bring together scholarship from STS and the wider social sciences to examine the ways in which actors, insiders and observers negotiate unbuilt and unfinished infrastructures – especially those where this unbuiltness or unfinishedness is a normal state of affairs. This work is analogous to an emergent framing that views STS as an analytical approach and the urban sphere as a strategic site for the study of infrastructure (Barry, 2013; Denis et al., 2016; Edwards et al., 2009; Graham and McFarlane, 2014; Mattern, 2018; Nucho, 2016).

This framing becomes even more evident when one looks at urban studies of the Global South. Here, scholars have gone as far as employing innovative concepts in their attempts to examine the dynamic and relational aspects of infrastructures shaped through their variegated interaction with urbanization. Focusing on everyday urban infrastructures, scholars have framed urban infrastructures as heterogeneous (Jaglin, 2014; Lawhon et al., 2018), hybrid (Furlong, 2014; Guma et al., 2019), incremental (Silver, 2014; Simone, 2008), and peopled – drawing on Simone’s (2004) famous work on ‘people as infrastructure’ that extends ‘the notion of infrastructure directly to people’s activities in the city’ (p. 407). These studies are effective at highlighting a variety of ad hoc actions and improvised mechanisms through which residents create possibilities of patchwork mechanisms that enable urban residents to connect to new infrastructural worlds. They are significant in the sense that they not only augment Star and Ruhleder’s relational in-practice approach to infrastructure within urban contexts, but also counter completist pursuits in the Global South. They make a great contribution in questioning the privileging of the LTS paradigm, the dominancy of a singular account, and the universality of an infrastructure network. In sum, these studies add to the knowledge reservoir that thinks through complex infrastructural socio-materialities, particularly those that continue to defy conventional theoretical logic. Most importantly, they invite us to discern infrastructures through their heterogeneity rather than universality, diversity rather than uniformity, and incompleteness rather than completeness.

### Engaging with the notion of incompleteness in the South

In the literature, we can find three ways in which the notion of incompleteness is used in connection with infrastructure. The first employs incompleteness in ways that project a negative value. Herein, ‘incompleteness’ is employed as a pejorative term to categorize ‘dysfunction’ (Choplin and Ciavolella, 2017: 325) or a ‘lacking in something’ (Ricci, 2015: 10). In other words, the notion reflects narratives of deficiency as opposed to normality. It portrays absence as opposed to presence. And more generally, it portrays how these translate into ‘inadequacy’ in Southern contexts (Chakrabarty, 2000: 32). In the second use, the notion of incompleteness is employed as an inevitable constitution of urban infrastructures in Southern postcolonial contexts. In this regard, incompleteness is used to denote ‘partial completion’ of the project of modernity (Graham and Marvin, 2001: 82; also Silver, 2015).

However, in the third use we find that incompleteness conveys a rather more creative and relatively balanced outlook. Urban and infrastructure domains of the South are thought of through their ordinariness, continuous ‘trajectories of incrementalism’ (Simone, 2008: 28) and different assemblages amidst ‘numerous and ever-changing rhythms of the city’ (Pieterse, 2008: 6). For instance, in his inventive work Simone employs the notion to demonstrate how infrastructures operate as a means of ‘passing on’, actively maintaining ‘a sense of incompletion’ (2016: 158). Simone captivatingly views the Southern city as a sphere where ‘everything [is] incomplete, shocked open, ready to be refigured, to pass on’, where ‘infrastructure is never complete’ (2016: 154–155), and where there is in fact, ‘a preference for keeping things incomplete’ (2014: 322–330). De Boeck, in his work in Kinshasa in the Democratic Republic of Congo, has a similar framing. Drawing attention to a kind of incompleteness in which material infrastructures of the urban fabric mediate boundless possibilities and affects, De Boeck (2014: 540) highlights a kind of ‘absence, lack and incompleteness’ that is shaped by ‘the daily rhythms of urban life’.

This framing aligns with Africanist philosophical conceptions where incompleteness is employed in an even more reframed manner to envisage an acquiescence to particular ways of knowing, being and becoming; and a necessary and celebrated condition present and evident in everything that exists. For example, Nyamnjoh (2015, 2017) draws inspiration from the works of prominent African thinkers, one of whom is Amos Tutuola. In *The Palm-Wine Drinkard*, Tutuola (1952) depicts the world as one of infinite possibilities where nothing is ever complete and where to quest for completeness is to be oblivious of the elusive and infinite reality of incompleteness. Tutuola’s tale, Nyamnjoh (2015) argues, more than being a mere work of fiction, reflects an African endogenous epistemology – one grounded in situated folklore and founded on the lived realities of the Yoruba in West Africa, where incompleteness is no more than an innate and present-continuous mode of being, a normal order of things.

These engagements are particularly imperative because they highlight the vitality of ‘incompleteness’ as an explanatory category in the social sciences. They highlight interpretations of ‘incompleteness’ as a notion that does not denote a predicament of missing something, but a never-ending state of becoming; it does not imply a condition that arises because of absences, but because of possibilities. Thus, these engagements draw us to a conception of incompleteness that is not necessarily a signification of something negative, but in and of itself a source of potency. They draw us to a type of incompleteness that is an inherent and ever-present condition of reality – as opposed to a strategy for completion. In the process, they echo what urban scholars including Bhan (2019) refer to as the ‘Southern mode of urban practice’ in which urban domains evolve with neither predetermined outcomes nor ultimate goals for seamless functioning or ubiquity. These engagements provide a crucial framing for operationalizing theoretical pluralism within infrastructure studies, here particularly by engaging with African studies. This is important because, in as much as STS and urban studies gives credence to alternate intellectual formulations, African studies have been de-valued within the hegemonic sphere of theory production and on several occasions described as metaphysical, spiritual and alternate belief systems that do not meet the standards of rationality and scientific rigor (see e.g. Mavhunga, 2014, 2017).

### Framing ‘incompleteness’ as a core feature of urban infrastructure

While the notion of incompleteness has featured on occasions in reference to urban domains and African contexts in the South, it still remains largely unexplored as a frame for discerning the nature of urban infrastructures in transition. Moreover, we still do not know much about how inside observers and actors (beyond scholars and authors) in the South make sense of their own interactions with infrastructure as incomplete. In this article, I recuperate ‘incompleteness’ from its material origins in the social sciences, particularly African philosophy, and extend its conceptual leverage and empirical value to STS. Hence, I employ the notion as an entry point for opening up questions around the nature and complexity of urban infrastructures, in line with STS debates that eschew monolithic frameworks synonymous with ‘technology determinism where technology is perceived to develop independently of society’ (Ward, 2005) and foreground ‘the locality of science and technology, and the consequent conﬂicts and other relations between localized science and technology’ (Sismondo, 2010: 196). I argue that the frame of ‘incompleteness’ adds to and extends wider articulations at the heart of the STS discourse, particularly those that examine ordinary technical devices (Akrich, 1993; de Laet and Mol, 2000) as constantly shifting, mutating and enduring in their trajectory over time (Barry, 2017). For this purpose, I identify and articulate distinct elements of *transiency, continuity* and *contingency*.

*Transiency* echoes configurations that materialize not due to standard or blatant situations of ‘failure’ – such as ‘when infrastructures cease to work’ in moments of disruption caused by technical or mechanical breakdown, or large-scale, stretched-out collapse (Graham, 2010: 3), including collapses that result from states failing to keep up (Anand, 2015; Nucho, 2016) – but due to the very nature of infrastructure as inherently transitory. It highlights a type of ephemerality that is not necessarily symptomatic of infrastructural damage and deficiency or fragmentation and incompetence, but an acknowledgement that infrastructures are always subject to temporality.

*Continuity* demonstrates the shifting, mutating and enduring nature of infrastructures at the interstices of constant repair, maintenance, and caretaking (Jackson, 2014; Mattern, 2018). It reiterates the actuality of incremental redefinition through continuous preoccupation of ‘tinkering’ (Knorr-Cetina, 1983), ‘patching up, reconfiguring, interpolating, and reassembling’ (Denis et al., 2016: 9) artefacts from previous forms to new forms of existence. It highlights the often-overlooked innovation practices (Jackson, 2014) of ‘those at the “receiving” end’ (Edwards et al., 2009: 371) – in addition to experts and system builders – who are constantly reinventing socio-technologies through a ‘kind of piece-by-piece adaptation and many small increments’ (Graham and Thrift, 2007: 5).

Finally, *contingency* points to how uses of infrastructures are not in fact fixed (Star and Ruhleder, 2001), but are products of assemblages that comprise of social, political, economic and technical negotiations – both trivial and significant, mundane and strange. It underscores new possibilities amidst uncertainty. It highlights the imperative of discerning heterogeneity beyond obvious surface irregularities, vulnerabilities and assumed accretions or corrosions of infrastructure (Anand et al., 2018; Barry, 2013; Graham et al., 2013; Jackson, 2014), and characteristically acknowledging infrastructures for what they really are – as emergent, shifting, and in that sense incomplete.

These elements are imperative for framing ‘incompleteness’ as a core feature, and even a virtue, of urban infrastructures in transition. I employ them as organizing concepts to learn with, from and about mobile infrastructures in the South. I employ them as ‘elements of incompleteness’, with the aim of destigmatizing occurrences of infrastructural heterogeneity, expanding framings of incompleteness, and providing an empirical and operational framework for discerning the construction, utility and design of urban infrastructures within a situated setting. I contend that these elements are imperative for understanding infrastructures that refuse to evolve as do their counterparts elsewhere, to serve the purposes for which they were envisioned, or to meet the demands for which they were employed.

## Scenes from the mobile age in Nairobi

**Figure 1.**
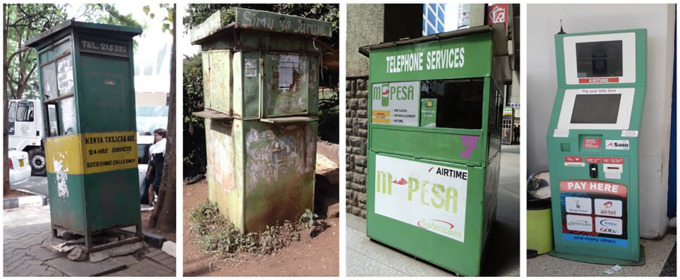
Incompleteness of mobile infrastructures in transition. Photos taken in Nairobi by author in 2016.

Although mobile telephony in Nairobi began in 1992, it was not until the late 1990s that its diffusion would take place. Prior to the 2000s, mobile telephony was marked by a bleak beginning. Fixed telecommunications networks, including telephone-booths were more prevalent during, while mobile phones were too expensive for the ordinary person to afford.^4^ Possession of a mobile phone (a typically over-sized device, sometimes the size of a standard police radiophone and usually heavy with a large retractable antenna) was often a statement of class and power. It is only by the early 2000s that Nairobi began to witness wide spread of mobile telephony (United Nations, 2007). Over the years since then, mobile telephony has become informed by exigencies of makeshift urbanism, bowing to the logics of incompleteness. This is partly evidenced by how it has largely materialized in the form of compact, mundane and emergent infrastructures such as kiosks, stalls and automated machines. Figure 1 above shows the empirical trajectory of mobile telephony as viewed through these kinds of infrastructures.

The images include, from left to right: a repurposed Telecom Kenya (one of Kenya’s integrated telecommunications providers) phone booth, a vestige of a Simu ya Jamii kiosk operated by Safaricom, an M-Pesa stall owned by Safaricom, and an automated kiosk operated by Solo Payment System, a private vending machine supplier. Such objects arrived at particular moments in the evolution of the mobile age in Nairobi. They occupy some of the most strategic spaces and premises of the city, including pavements of its downtown alleys, corridors of its extensive shopping malls, and narrow paths of its shantytowns. As one of my respondents in Soweto-Kayole indicated, these infrastructures have become ‘part of what constitutes our technological culture’. The spatial and sensory aspects of how they are experienced within these areas shape the public culture of Nairobians around what the mobile age actually represents or looks like.

During my fieldwork, I observed that most of these infrastructures were evolving not as clearly neat structures, but rather as vestigial arrangements within messy urban realities. In an aesthetic sense, these infrastructures appeared far from seamless functioning, precision or completion. Because of their very nature as excessively patchy and segmented, from the standpoint of an outsider, these infrastructures might reveal a sequence of objects limited by resources. Moreover, they might reveal a failed imitation or translation of circulating ideals and technologies of the mobile age. However, below I argue that these infrastructures represent successive periods in the development and growth of mobile telephony. I do so with the aim of learning with, from and about mobile infrastructures in the South. I demonstrate how and in what forms ‘incompleteness’ becomes a salient feature of infrastructure development for insiders and observers. I provide evidence to the fact that the insiders in Nairobi do not necessarily regard them as lacking or wanting, and certainly not as dysfunctional, but rather as constituting a normal order and accepted – or even preferred – condition of urban life.

### Configurations of transiency

The foremost Simu ya Jamii kiosks in Nairobi were introduced in 2003 by Safaricom, through a partnership with South Africa’s phone company, Adtel. In their design, kiosks borrowed heavily from ‘telephone boxes’ of the 1980s and ’90s (see the first image in Figure 1 above), which were often operated by Telecom Kenya, a leading provider of landline services in the country at the time. In Nairobi’s central business district, as elsewhere, these boxes were essentially coin-operated and originally yellow in color. They were typically assembled as fixed metallic ‘telephone boxes’ that connected parts of the entire country through an electricity-like network of pole wiring. As they were phased out, some of their remnants (including the one shown in the first image of Figure 1 above), became repurposed and refurbished.

While Simu ya Jamii kiosks’ design and material form drew strongly from ‘telephone boxes’, especially in the sense of a being built structures typically nesting public pay-phones, their rise marked the transition from the corded ‘telephone boxes’ to mobile phones. As plainly put by a local critic and resident in Soweto-Kayole, ‘they were the bridge between the “Call-Box and PO Box era” to the era of the mobile [phone]’. They encompassed both mobile and fixed features. Because of this, they foreclosed the idea of mobile phones as entirely mobile in the typical sense of a portable, movable and flexible device – colloquially referred to in Kenya as *simu ya mkono*. They were a quasi-mobile fixed technology whose materialization took liminal forms. As such, they are commonly said to have played a significant role in preparing the minds of Kenyans for the mobile age.

Additionally, the kiosks were transient and assembled out of makeshift, patched, fabricated and repurposed materials – perhaps resulting in something like a wooden shack with a tin roof. For the most part, the kiosks were green in color and highly mobile both in their form and operation. As I observed during my fieldwork, sometimes they were placed besides or in the place of the often-vestigial corded telephone box. Their walls often would be painted green or, in other cases, multiple layers, one on top of the other, but ultimately with the green color on top. Frequently, the kiosks would be installed in the busiest and most strategic public spaces, such as in the alleys of informal markets.

Moreover, Simu ya Jamii kiosks were also communized both in their ownership and operation. They were unveiled as community artefacts and were designed to operate as community-based kiosks. As community-based, the kiosks challenged the definition of a mobile ‘individual’ handset. They essentially foreclosed individual forms of phone ownership. Instead, they fostered and sustained communal forms inherent in the very operation of phone kiosks. By extension, they became the kind of makeshift infrastructural elements that enabled – and were enabled by – situated notions of communality and sociality. For example, communal practices in the use and appropriation of the Simu ya Jamii kiosks are embedded in the people’s vernacular arrangements founded on notions of *chama* (collectivism)^5^ and *harambee* (distributionism).^6^

Simu ya Jamii accommodated notions of mobile phone sharing amidst realities of low telecommunications density in Nairobi. Different patchwork strategies were employed through different notions of phone sharing. The first notion, invented by Safaricom, enabled flexible ownership and use through relatively subsidized tariffs and flexible billing options. The second, however, was resident-initiated and based on a mode of practice where residents in the proximity would actively invest labor, care and maintenance into the daily operation and sustenance of the structures. For instance, those who lived or worked closest to a kiosk took on the responsibility of protecting the structure against vandalism and subversive usages. Sometimes, they were tasked as custodians, providing security. In this case, these infrastructures would begin to shape a specific kind of politics in which power was redistributed within the community that surrounded this technical object, an object that had to be operated, maintained and protected. These kinds of politics would lead to the creation of community-level and street-level bureaucrats, who became key nodes of power in the community with respect to the systems.

These arrangements intensified as Simu ya Jamii kiosks further evolved and became more marketable for urban residents, with the structures soon becoming a profitable business for them. For instance, the residents I interviewed in Mathare mentioned how beyond the usual hype of Simu ya Jamii in urban Kenyan settings, running a kiosk ‘was then a good and booming side-business’, ‘a good source of extra income’, and ‘generally, a good side-hustle at the time’. However, as the residents insisted, setting up a Simu ya Jamii kiosk as a business venture was not always straightforward. For example, it required a series of tedious procedures and actions, on top of exorbitant fees. In their recollections, residents in Mathare explained to me that an enterprising individual would first and foremost have to find – or claim – a space for a Simu ya Jamii kiosk. After that, the person would have to invest about KES 8,000 (USD 800) to KES 10,000 (USD 1000) to construct the kiosk. Then about KES 500 (USD 50) to KES 600 (USD 60) would have to be spent in buying the mobile handset and charger, and up to KES 400 (USD 40) in buying the SIM card. This card would have to be activated and required a monthly service fee for it to function. To meet these costs and break even, residents claimed that they often pooled money together. For instance, one of the residents in Mathare recalled:

We shared costs of use and repair and maintenance as a community. It was easier this way. There was no incentive for us to purchase personal phones. We preferred collective ownership as a community. That’s what everybody went for. Phone sharing was the thing at the time.

This practice of sharing costs became a way for urban residents to profit from the new technology without having to individually spend inflated sums. In response, Safaricom began to facilitate micro-ownership business strategies enabling small-scale entrepreneurship and investment. These strategies were enabled by different kinds of credit arrangements aggressively aimed at promoting such developments. Simu ya Jamii kiosks began to operate as business operations, particularly through such cost-sharing mechanisms. Sometimes, operations were driven by private enterprises, non-government and community-based organizations and technology companies where these groups and institutions would develop new modes and technologies of producing and distributing services in an open and incomplete infrastructure landscape.^7^ In Kibera, for instance, as the residents enumerated, a precedent emerged where collectives shared costs of ownership based on mutual interest, and community and trust, to afford the expensive gadgets. For residents in the low-income areas, it became the standard way for them to underwrite collective outlooks in developing and appropriating the Simu ya Jamii kiosks by themselves.

The more the kiosks became profitable for business, the more they lost their inherent communal appeal in places like Kibera. The kiosks increasingly became the source of a new culture of small-scale entrepreneurship, this culture becoming an unintended consequence of Simu ya Jamii. As a result, in several communities, new sociotechnical inequities, power dynamics and micropolitics created tensions between the individualistic and the communal elements of the technology, mirroring a clash of private and public culture. Take the example of Mathare: The residents in the settlement recalled that they did not particularly like the idea of Simu ya Jamii kiosks as personalized businesses. They argued that this idea did not fit into their own ways of life and that it was incredibly hard for them to adjust to the new corporate culture that had become synonymous with the kiosks. For many, the commodification of the kiosks went against the traditional practices of collectivity to which many in the community had been inclined. This became the main source of sociotechnical conflict for Simu ya Jamii: An infrastructure which, while originally designed and deployed by Safaricom as a public-communal artefact, was turning into a private-entrepreneurial venture.

Transience, as we have seen here, is a vital element of incompleteness because it reflects the inextricable avant-garde of ostensibly patchy, mundane and ephemeral infrastructures in transition, but also by the everyday survivals that these infrastructures portray. It highlights a type of ephemerality that acknowledges infrastructures as inherently temporary, subject to resident-initiated practices and processes which impose makeshift and transitory boundaries. As shown, Simu ya Jamii kiosks exemplify a type of incompleteness that is representative of immense potential – especially in their consideration of the subaltern and transient practices of organic, vernacular and improvised urbanism – as opposed to something ‘rendered unfinished’ (Ricci, 2015: 10). As such, they constitute a type of ‘tactical’ institution (Simone, 2016) that functions through makeshift processes. These processes are backed by insiders’ creativity and laborious *tinkering* (Knorr, 1979; Knorr-Cetina, 1983) as a way of making Simu ya Jamii kiosks fit their own particular settings and contexts. They resonate with users’ typically unscripted ways of getting by and making use of ‘what is available now’ within urban worlds in which adversity and scarcity are the norm of life.

### Continuities in transitioning regimes

By the late 2000s, most of the Simu ya Jamii kiosks had been phased out. In their transience, they had succumbed to ephemerality: here today, gone tomorrow. To paraphrase one resident in Soweto-Kayole, the ephemerality that had birthed them had ended them. Besides being ephemeral, their disappearance had to do with the new competitors.^8^ Chinese mobile phones had entered Kenya’s urban market, making it increasingly possible for the ordinary resident to afford a personal mobile handset. As a result, Simu ya Jamii operations gradually diminished, eventually coming to an end. Many kiosks were either highly isolated or deserted. Many others were subjected to decay and collapse in the face of surface indiscretion and precarious accretion, as narrated by one community representative of Soweto-Kayole, a low-income suburb in Nairobi:

Many of the kiosks became unpopular. … Some were unkempt. Others were vandalized. Many were in a poor state. Passersby used them as disposal points during the day, and lavatories in the night. [But mostly] handling and maintaining them in some places proved costlier especially for owners who [with time] were then asked to acquire permits which cost hundreds to thousands of [Kenyan] shillings. Those who owned the kiosks were forced to hike their charges [for pay-phone use] if they were to stay viable. This made [Simu ya Jamii] very expensive – even for the users. These frustrations made individual cellphones popular and replaced the community ones.

In the post-Simu ya Jamii era, many of the Simu ya Jamii kiosks were a small remnant of what had formerly been a ubiquitous infrastructure, representing a spectral presence, a shadow of their own self. Freed from their past, Simu ya Jamii structures took on a different life, becoming metasomatized. As metasomatized entities, Simu ya Jamii kiosks begun to function in a different capacity. They became the subject of further conversion in the typical sense of something still not yet complete. As such, they evolved into something else: They powered M-Pesa, a successor technology for electronic money transfer and payments.

As M-Pesa stalls, they facilitated money transfers, cash conversions to mobile money, deposits, savings, microfinance disbursement and remittance delivery. Besides acting as mobile providers’ dealerships and non-bank entities, the agents and vendors who operated the green structures also handled customer registration for new users and met the liquidity needs of consumers. They also served as connection points for bank-related financial transactions and utility-related bill payments. In addition, as unintended usages or interpretations, the M-Pesa stalls served as everyday convenience shops for basic items such as phone chargers, batteries and other interchangeable parts. In other cases, they also served as ticketing points for the city county officials and as public lottery access points for betting and staking services.

As incomplete infrastructures building on ones that had preceded them, M-Pesa stalls, very much like Simu ya Jamii kiosks, were also distributed in the city, often located along some of the busiest and most strategic spots of its outskirts. They were oftenfound in prominent places and positions in the outskirts of the city’s most recognized streets and markets, adjacent to where people worked, or in the informal spaces where people lived or settled. Still, like Simu ya Jamii kiosks, M-Pesa stalls served additional purposes as advertising displays for the mobile service provider. The stalls were often green in color, representing Safaricom. From my viewpoint as an outside observer, these structures sometimes appeared as somewhat undesirable and in want of – or at least lacking aspiration for – aesthetic splendor. But this was not the case for those who inhabited the spaces in which these structures were located and who saw their value beyond appearances and aesthetics. Indeed, the spatial zones of these structures were often communally securitized by intermediary agents who acted as custodians or consigns – just as was the case for Simu ya Jamii kiosks.

In Kibera, the residents that I interviewed believe that Simu ya Jamii paved the way for the rise of M-Pesa. Simu ya Jamii was remembered for being ‘famous in the days’, particularly for having shaped small-scale entrepreneurship and mobile airtime credit vending. It still held a significant place in the social and business culture of the community, albeit not so much of an iconic novelty as it had been in the early 2000s as a Simu ya Jamii. The reinvented stall, like many in Nairobi, holds historic, architectural and cultural value – both as one that evolved over time as a new modality of access to mobile telephony and sustained communal ways of urban life. The stall remains quite colorful, explicit and marked in pen and spray paint with fading words ‘Simu ya Jamii’, underneath new marks of ‘M-Pesa’. Huge advertising posters could still be seen to be glued and affixed to the sides of the kiosks. Through the continuities of different rationalities, the stall can be seen as representing an authentic continuation of a transitioning regime. This structure reflects how, in their obsolescence, Simu ya Jamii kiosks lived on through M-Pesa stalls, as one of my respondents, a Kenyan blogger and journalist in Nairobi, explains:

This is Nairobi, we do not set bygones to rest. We capitalize on them. …Simu ya Jamii lives through M-Pesa.

As such, within Nairobi’s urban milieu Simu ya Jamii kiosks are considered to have been highly prototypical as crucibles of sociotechnical innovation. The same could be said of M-Pesa. One example that further attests to their prototypical nature – and incompleteness in the same breadth – is the emergence of automated kiosks such as those operated as Solo Payment System (in the fourth image in Figure 1). The automated kiosk appears to be a technological modification of the Simu ya Jamii kiosk and M-Pesa stall, particularly in its aesthetic resemblance to them. Moreover, as vending machines, they are also commonly found in spaces within some of Nairobi’s large shopping centers and along the wide streets of the city. Automated kiosks provide all functions of the M-Pesa stalls^9^ – the only difference between the two being that the M-Pesa stalls are not automated and require physical labor or agents to attend to customers.

Continuity as an element of incompleteness demonstrates the shifting nature of infrastructure. From Simu ya Jamii kiosks to M-Pesa stalls, the continuities reflect how, technologies not only mutate but also endure, clearly evolving into something else. They reflect emergent infrastructures as incessant in their flexibility to convert and change into something else; never completely dying but alterating to a different form. Thus, M-Pesa and Simu ya Jamii reiterate the actuality of incremental infrastructural redefinition as a continuous piece-by-piece adaptation, often shaped by more inclusive and situated innovation practices and sociotechnical processes (see e.g. Edwards et al., 2009; Jackson, 2014). They embody possibilities for change and modification as clearly reflected by the landmarks I recorded in my field notes:

Phone-booths, Simu ya Jamii kiosks, M-Pesa stalls, automated kiosks. Former phone-booths, now Simu ya Jamii kiosks. Former Simu ya Jamii kiosks now M-Pesa stalls. Old structures evolving into new ones. The old structures and the new ones existing side by side, both retaining their unique aesthetics …. The new ones never completely replacing the old ones. The old ones refusing to completely discontinue. … both complementing each other. The last building on the footprints of the one before it. All building on the legacies of their predecessors.

### Contingency in the face of encounter

Beyond its continuities with Simu ya Jamii, the incompleteness of M-Pesa is best viewed when one considers its contingency in the face of encounter. M-Pesa was piloted by Kenya’s leading mobile service operator, Safaricom, in October 2005, as a branchless-banking service for women’s cooperatives. However, whether out of subversion or convenience, M-Pesa developed far beyond its original scope to portray a nomadic style of spiraling outwards.

In its evolvement, M-Pesa opened up to further incremental improvisation and progressive widening with its initial deployment, unearthing unanticipated usages from users who were determined to inscribe other functions into the technology. As such, M-Pesa evolved beyond its original concept as a monthly microfinance loan disbursal and withdrawal. It evolved to become a service that facilitated money transfer and remittance delivery, served as a virtual repository of cash, and was a virtual payment system for goods and services through auxiliary platforms such as Lipa na M-Pesa (Pay with M-Pesa); especially this last service enabled it to become a significant element of everyday urban activities. Table 1 below illustrates how M-Pesa has undergone continual redefinition through a process in which its infrastructural logic has gradually modified and transformed in the context of Nairobi.

**Table 1.** Incompleteness of M-Pesa.

Microfinance	2005	M-Pesa officially launches as a monthly microfinance repayment service. Commences as a small-scale pilot program in three locations: Thika, Mathare and Nairobi Central Business District.
	2006	M-Pesa ends pilot. However, it stays in service for targeted users who had re-appropriated and domesticated its function from microfinance repayment to money transfer.
Money transfer	2007	Safaricom re-launches M-Pesa. Readjusts technology from microfinance to mobile money transfer, capitalizing on a simple value proposition with a core money transfer function: ‘send money home’.
Bill payment	2008	M-Pesa expands services beyond mobile money transfer, toward bill payment, opening up to new encounters.
	2009	For example, urban water and electricity agencies (including Nairobi City Water and Sewerage Company, and Kenya Power and Lighting Company) build partnerships with Safaricom to enable mobile payments for utility services.
	2011	Also, private firms such as M-Kopa Solar seek partnerships with Safaricom for mobile-based purchases for products and services, including payment and crediting systems.
Payments for goods/services	2013	Safaricom launches Lipa na M-Pesa as a more urban mobile payment service for payment of utility bills and other purchases.
	2015	Safaricom runs campaigns to entrench Lipa na M-Pesa, providing incentives for new merchants and vendors such as supermarkets and petrol stations.
	2016	Safaricom attempts to further upgrade M-Pesa’s technological infrastructure, testing a new card-based method of payment linked to Lipa na M-Pesa.

Table 1 illustrates M-Pesa’s progressive widening (and to some extent narrowing) as a technological product that has constantly changed and been redefined without mandatory predetermined outcomes nor ultimate ambitions for achieving ubiquity. This (widening and narrowing) has been contingent on different factors. The first concerns the political and economic conditions that led to the strategic visibility of M-Pesa. This factor is encapsulated in what has come to constitute one of the great ironies of M-Pesa’s rise. While originally conceived in 2005, M-Pesa did not gain much social and material visibility until 2008, when Kenya was plunged into moments of turmoil during the 2007-08 presidential election, when evidence of vote-rigging emerged. During this period, infrastructures such as roads, railway, banks and public transport had either collapsed or become disabled. The intensification of violence had significantly restricted physical flows, human mobility and accessibility of services, especially in the city’s opposition strongholds, such as the notorious informal areas like Mathare and Kibera. The residents in Kibera told me that during this time goods and services could not be physically moved into the settlement from across the many areas of the city. Kibera, like many parts of Nairobi, was isolated, as many of the roads were blocked or blockaded, while parts of the railway line were either destroyed or disassembled. The banks and other financial institutions remained closed and inaccessible. Left with no other choice whatsoever, urbanites reverted to M-Pesa – as one of the residents in Kibera described her experience to me:

That was the first time when I realized that I could use my phone for ‘mobile money’. I was distressed by the conflict in the city. I remember calling my family back in the village asking if through the new service that Safaricom had installed, they could send me money by mobile phone. I received an SMS notification within an hour indicating that I’d received credit on my phone.

While M-Pesa in Nairobi had had a very tenuous presence, and held limited to no value for many urbanites, the uncertainty of the city’s violence animated a desperate need for money from those holed up in their residences needing to escape the warfare. This uncertainty also galvanized those who sought to cope in the midst of the temporary closure or inaccessibility of physical banks. Many residents, for instance, recounted how M-Pesa became their provisional bank. They also recounted how M-Pesa provided a primary platform for branchless banking in those dissonant times. In the absence of road and railway connections and access, M-Pesa provided mobile-based connectivity and credit accessibility to those who were unable to move and desperately in need of pocket change to survive. Some residents said that in the aftermath of the violence, they continued to use the platform out of convenience for their financial services. Hence, they would begin to see their mobile phone as a safe haven for such purposes. In other words, the violence and turmoil in Nairobi had shaped M-Pesa’s growth and visibility.

The second factor has to do with the fact that M-Pesa was greatly marked by an invisible state and the absence of overt state regulation.^10^ The state constituted a mode of politics synonymous with ‘hanging in there’, ‘playing along’ and ‘keeping away’ – as opposed to succinctly setting a policy agenda for the new technology. As has been recounted by the experts and system developers in different fora, the state’s supervisory authorities, including the National Treasury and the Central Bank of Kenya, mostly sought to create policy as a response, rather than a strategy. As such, M-Pesa typified ‘a classic case in which innovation preceded policy’ (Ndemo, 2016: 356). The government often appeared to not know how to respond to the technology’s increased transformation. Being an emergent technology, M-Pesa did not attract much interest from bureaucrats or political elites. Many in the government considered it to be on a technical terrain, only for technicians. They located M-Pesa outside the realm of politics, with the state refraining from overtly interfering with its operations.

This was the case for its system builders and technicians as well. They, as Michaels (2011: 7) reports, adopted a ‘watch and learn’ approach, not overtly interfering in the system’s growth. Inherent in this approach was the vitality of the users, who played an integral role in this process as new experts stepping front and center, while system developers reverted to playing catch-up. One of the technology developers at I-Hub, a leading technology hub and incubator in Nairobi affirms this:

The system upon which M-Pesa operates today typically grew out of user-led practices. M-Pesa did not enter Kenya in a stabilized form. System builders, whether they labor to make it clear or not, followed the lead of the people. Kenyans were not mere consumers in the development of M-Pesa; neither were they mere participants in it. They were the experts of the whole thing.

M-Pesa’s developers, while aware of the technology’s need for continuous expert attention to ensure technical capacity and periodic updating, played second fiddle in ‘letting things be’, ‘watching closely’ and ‘leading from behind’. Safaricom offered agency to the users through its active (albeit silent) role, as a technology developer. As one technology expert in Nairobi with experience and knowledge of the workings of M-Pesa pointed out:

Safaricom did not entirely view M-Pesa as an end in itself. Neither did they view it as an absolute means to a predefined need. They demonstrated restraint in the purpose that the technology could serve and understood the local knowledge systems and practices. They endeavored to make the technology work by meeting cultural and socioeconomic contexts.

The above quote speaks to the incompleteness of M-Pesa. It was the result of a concerted and devoted institutional impetus to embrace users’ evolving preferences, expectations and practices. It was part of an impetus where, rather than simply privileging select experts or models of how the technology should evolve, the developers paid overt attention to socioeconomic circumstances surrounding its customization and domestication, and listened to the end user. These processes served as a source of potency for M-Pesa’s sprawl, exposing it to possibilities sustained through its material conditions of contingency.

Going by the narratives from strategists and experts in Nairobi’s tech-scene, M-Pesa’s developers continuously modified the technology without necessarily compelling a predetermined outcome for it. The developers allowed a series of incremental re-adaptations and structural modifications through which M-Pesa morphed over its initial years. They exhibited a preference for keeping the technology incomplete and open to possibilities of change and adaptation. In so doing, they desisted from imposing their own views of how M-Pesa should look or function. As such, they recognized the active role of the users, and the micropolitics in negotiating the technology to make it serve their own ends. M-Pesa has remained incomplete and in a constant state of transition. It has remained a technology that still could be modified and reconfigured to fit wider or more specific dynamics of the space and time.

In the context of contingency as an element of incompleteness, the account here is valuable for two reasons. First, it shows that infrastructures are built not from scratch by their system-builders, but through cooperated processes by the different stakeholders, who include end-users. But more importantly, it takes away the assumption of finality in the making of infrastructures – or the notion that there is such a thing as a final or complete product. Thus, it points to how infrastructures are not in fact fixed, black-boxed or neutral, but are products of political, economic, and sociotechnical negotiation – both trivial and significant, mundane and strange. It underscores new possibilities amidst uncertainties, and characteristically acknowledges infrastructures as always incomplete.

## Conclusion

In this article, I have made the case for ‘incompleteness’ as a core feature and virtue of urban infrastructures in transition. In making this contribution, I have drawn from the experience of successive periods in the growth and development of infrastructures of the mobile age in Nairobi – including the mobile infrastructures of Simu ya Jamii kiosk, the M-Pesa stall, and the M-Pesa platform – to bring to the surface *transiency, continuity* and *contingency* as ‘elements of incompleteness’. As I have argued, when seen through these elements, the infrastructures studied make three complementary arrangements salient.

The first arrangement is one that portrays infrastructures in transition as transient in nature. This transience is epitomized by the specific purposes that infrastructures in transition serve, the specific groups of people they serve, and the specific temporalities in which they are serving. This is exemplified by the Simu ya Jamii kiosk’s lasting only a short time. The second arrangement is one where emergent infrastructures, when seen through their succession from one regime to another, do not appear to be entirely new or different from their predecessors. Instead, they constitute elements of the old that provide the pedestal – and ease the way – for the ‘new’, as seen in the case of the M-Pesa stall, which reveals the continuation of a preceding transient regime as a technology that takes on the reigns of the Simu ya Jamii kiosk. This arrangement, in Southern urban infrastructures, is also articulated not least by Simone, who demonstrates how old regimes do not always necessarily die or yield even in their obsolesce, but continue to operate as a means of ‘passing on’ (2016: 158) particularly in ways that reflect active and purposeful incompleteness. The third arrangement is one where urban infrastructures in transition can be viewed as characteristically contingent and possessing no precise intentions for appearing complete or arriving at a complete form. This is best revealed by the M-Pesa platform, a technology that is constantly being modified, enduring over time yet remaining incomplete. These arrangements highlight the imperative of examining Southern infrastructures not just as empirical conduits, but as entry points to theorizing and understanding infrastructural heterogeneity and diversity. They demonstrate the imperative of transcending homogenizing accounts and fixations of how infrastructures in general, and Southern infrastructures in particular, should actually look like or function.

So, this article provokes a revisiting of infrastructure systems through a new and alternative lens, beyond North-centric vocabularies of success and failure. It invites us to rise above the language of ‘completion’ which presumes incompletion as a state in which the infrastructure has not yet been achieved or remains unattainable. Likewise, it draws us to the conceptual contribution of ‘incompleteness’ as a notion that allows us to escape presuppositions about inadequacy, or any possibility that the idea of incompleteness is predicated on failure or lack. In this regard, ‘incompleteness’, as I have posited here, goes further than the already quite nuanced approaches that examine ordinary technical devices or objects in a fluid sense as entities embedded in collective conventions and structures of everyday life. This is evidenced by my endeavor, which integrates insights from Africanist philosophy, the Southern mode of urban practice, and empirical findings from my own research in Nairobi, to make salient the nature of infrastructure as emergent, shifting, and in that sense incomplete.

As an analytical approach, ‘incompleteness’ provides a more explicit framework for discerning infrastructural processes which, while diverse and heterogeneous, cannot be described as failed or fragmented, because they are something else entirely. Its empirical value lies in the fact that it draws us to a new way of seeing urban infrastructure as composed of entities that contain an innate and present-continuous mode of incompleteness. It serves as a reminder of the need for further situated and located engagement that transcends homogenizing solutions to processes of infrastructural heterogeneity, and provides an alternative to unidimensional understandings of infrastructure development that foreground lack and failure. As such, ‘incompleteness’ counters selective accounts that disparage diverse and heterogeneous infrastructures. It prompts a revisiting of what we think we know – or view as familiar or strange – about urban infrastructures in transition. It transcends completist frames and offers a carefully restrained form of theoretical relativism in discerning (occurrences of) infrastructural heterogeneity and diversity in the urban South and beyond. But most importantly, it challenges us to step back and think differently about infrastructural processes that do not ‘yield’ or conform to dominant standards or norms. Thus, ‘incompleteness’ takes us back to the foundational question of teleology within infrastructure studies, particularly concerning what a complete infrastructure even looks like.

## Notes

1. Many studies of infrastructure are often concerned with the most significant innovations or infrastructures perceived to command enormous stature or impact, to the detriment of liminal and less valued infrastructures. As Morphet argues, the world has become ‘so overloaded with big stories and important information that the small and peripheral have come to seem a positive value’ (2006: 86). Inquiry needs to open up to these small and marginal infrastructures which provide new opportunities as crucibles for radical new socio-technical paradigms. Small and marginal infrastructures have a prominent role to play in theorizing. They have the potential to illuminate not just peculiarly of situated experiences, but also to incite wider questions about alternate modernities and material conditions of the South.

2. In this paper, I define infrastructure as that which is essentially embedded in the collective conventions and structures of everyday life, so much so that while inherently lively and visible, might sometimes be rendered invisible or other times taken for granted (Star and Bowker, 2006). My definition of the notion therefore is fluid. It encompasses elements that would in some cases be referred to by other labels: For illustration purposes, we could take the work of Akrich (1993) on the photovoltaic kits developed by French industrialists for the developing world market, including Africa, or de Laet and Mol (2000) on the bush-pump specifically designed for African contexts like the Zimbabwe, which both beautifully examine ordinary technical devices or objects. The Simu ya Jamii kiosks, M-Pesa stalls and the M-Pesa platform might fall within the same category – as technical devices or objects. However, I employ the term ‘infrastructure’ cognizant that category distinctions between object, device, and infrastructure are fluid.

3. In *What do Science, Technology and Innovation Mean from Africa?* Mavhunga (2017) demonstrates how within STS/I debates, Africa is often regarded as a recipient of theory and technology rather than a maker of them. It is imperative, Mavhunga argues, to not merely view Africa as a product of ‘technology transfer’ from elsewhere but also as a site of knowledge and technology production. Mavhunga rightly describes Africa as creative, technological and scientific. I follow this and similar calls for not only extending STS kinds of analysis into Southern contexts, but also for theorizing (technologies and infrastructures) from the South.

4. It remained an exclusive preserve of the high-class citizen, the wealthiest, and the ‘elite’ in high income areas of the city with prices ranging from KES 100,000 (USD 1000) and KES 300,000 (USD 3,000).

5. ‘Chama’ is a Kiswahili word that refers to a group of people with a common interest in coming together. The groups can be anywhere from two to more than 100. The meaning of this word is broad and includes both formal (i.e. registered associations and political parties), semi-formal (i.e. societal and community-based initiatives) and non-formal groupings (i.e. ad hoc collectives).

6. ‘Harambee,’ a Kiswahili word means ‘let’s pull together’. It originally referred to a traditional habit among societies to contribute their energy and time for communitarian work. The meaning and use of Harambee has shifted over time from national cohesion, to societal and communitarian contributions.

7. The other forms included: hawking in which mostly youth would forgo the shack or kiosk and carry handsets on lanyards around their necks and stroll around towns, villages and marketplaces in search of customers, and use of bicycles – that is, bicycle phones which were effective in the sense that they increased access in parts with low- or non-accessibility.

8. These include Celtel’s ‘Jembi Payphone’, KenCell’s ‘Simu Yetu’ (Our Phone) to Telekom Kenya Limited’s ‘Mzalendo’.

9. These include mobile payment services, such as water and electricity bills, mobile phone top-up purchases and the like. By the time of my fieldwork, they had become the ICT of choice for many residents of Nairobi preceding Simu ya Jamii kiosks and M-Pesa stalls had to meet the architectural regulations.

10. The state’s flexibility toward informality and improvisation in the appropriation and diffusion of mobile telephony innovation is said to have spurred the mobile money transfer network system to its present-day success. The Kenyan government, especially Mwai Kibaki’s administration between December 2002 and April 2013 and Uhuru Kenyatta’s administration since April 2013, have taken numerous steps and strategies to increase the adoption of new technologies in general and mobile telephony in particular. Attempts from the public sector have made it easier for M-Pesa and the mobile money transfer infrastructure to deeply penetrate the urban areas and beyond.
